# Comment on: Shortening surgical training through robotics: randomized clinical trial of laparoscopic *versus* robotic surgical learning curves

**DOI:** 10.1093/bjsopen/zrab030

**Published:** 2021-08-23

**Authors:** I Minty, Z Nowinka

**Affiliations:** Department of Surgery and Cancer, Imperial College London, London, UK; Department of Surgery and Cancer, Imperial College London, London, UK

*Dear Editor*

A recent study conducted by Gall *et al.*^1^ has shown to shorten surgical training through robotics. With an increasing demand for minimally invasive surgery, robotics could provide a valuable alternative to laparoscopy, which has a lengthy learning curve.

We would like to congratulate the authors on the study design and the choice of outcome measures. The study was well-powered and the Global Rating Score, and the Van Sickle assessment are both recognized, reliable and validated scoring systems.

In the surgical trainee group, the robotic group outperformed the laparoscopic group in seven out of eight domains. Laparoscopic approach was only superior in the time taken to complete the gallbladder and liver bed resection. This finding is impressive considering the surgeons had previous experience in performing laparoscopic cholecystectomy, which would presumably give that group an advantage.

The superiority of robotics was also convincing in the surgically naïve, medical student group. The use of robotics resulted in better quality, speed and absence of errors. However, the assessment took place the same day, or the day after the teaching, which limits the long-term inferences that can be made. The study would have benefited from further staggered assessments to evaluate the retention of knowledge.

Interestingly, in both laparoscopic and robotic groups, the medical students were regular video games players. Previous experience in playing video games might be advantageous when learning skills requiring excellent visuospatial awareness, such as robotic surgery. Although there was no significant difference between the two groups, this factor could have confounded the true gravity of surgical skills acquisition.

Nevertheless, the success of the medical students provides evidence of the benefit of introducing simulators earlier in training. The authors have provided an impactful study that could potentially alter the way surgical education is delivered.

## References

[1] Gall TMH , Alrawashdeh W, Soomro N, White S, Jiao LR. Shortening surgical training through robotics: randomized clinical trial of laparoscopic *versus* robotic surgical learning curves. BJS Open 2020;4:1100–1108 33052038 10.1002/bjs5.50353 PMC7709379

